# Mucoadhesive *Bletilla striata* Polysaccharide-Based Artificial Tears to Relieve Symptoms and Inflammation in Rabbit with Dry Eyes Syndrome

**DOI:** 10.3390/polym12071465

**Published:** 2020-06-30

**Authors:** Minal Thacker, Ching-Li Tseng, Chih-Yen Chang, Subhaini Jakfar, Hsuan Yu Chen, Feng-Huei Lin

**Affiliations:** 1 Graduate Institute of Biomedical Engineering, National Taiwan University, No.49, Fanglan Road, Daan District, Taipei 10051, Taiwan; minal.thacker11@gmail.com (M.T.); r05548004@gmail.com (C.-Y.C.); subhaini@yahoo.com (S.J.); hychen83@gmail.com (H.Y.C.); 2 Graduate Institute of Biomedical Materials and Tissue Engineering, Taipei Medical University, No. 250 Wu-Xing Street, Taipei 11031, Taiwan; chingli@tmu.edu.tw; 3 Institute of Biomedical Engineering and Nanomedicine, National Health Research Institutes, Miaoli County 35053, Taiwan

**Keywords:** *Bletilla striata* polysaccharide, dry eye syndrome, artificial tear, anti-inflammatory

## Abstract

Dry eye syndrome (DES) is a multifactorial disorder of the ocular surface affecting many people all over the world. However, there have been many therapeutic advancements for the treatment of DES, substantial long-term treatment remains a challenge. Natural plant-based polysaccharides have gained much importance in the field of tissue engineering for their excellent biocompatibility and unique physical properties. In this study, polysaccharides from a Chinese ground orchid, *Bletilla striata*, were successfully extracted and incorporated into the artificial tears for DES treatment due to its anti-inflammatory and mucoadhesive properties. The examination for physical properties such as refractive index, pH, viscosity and osmolality of the *Bletilla striata* polysaccharide (BSP) artificial tears fabricated in this study showed that it was in close association with that of the natural human tears. The reactive oxygen species (ROS) level and inflammatory gene expression tested in human corneal epithelium cells (HCECs) indicated that the low BSP concentrations (0.01–0.1% *v/v*) could effectively reduce inflammatory cytokines (TNF, IL8) and ROS levels in HCECs, respectively. Longer retention of the BSP-formulated artificial tears on the ocular surface is due to the mucoadhesive nature of BSP allowing lasting lubrication. Additionally, a rabbit’s DES model was created to evaluate the effect of BSP for treating dry eye. Schirmer test results exhibited the effectiveness of 0.1% (*v/v*) BSP-containing artificial tears in enhancing the tear volume in DES rabbits. This work combines the effectiveness of artificial tears and anti-inflammatory herb extract (BSP) to moisturize ocular surface and to relieve the inflammatory condition in DES rabbit, which further shows great potential of BSP in treating ocular surface diseases like DES in clinics in the future.

## 1. Introduction

Dry eye syndrome (DES) is one of the most prevalent ocular surface diseases in the worldwide affecting 8% to 14% of the population [1,2]. The aging population in Taiwan leads to the increase in the prevalence of DES. DES predominates in females more than male of all age group and the likelihood of developing DES increases with age [3,4,5]. It also generally appears in patients after cataract surgery and laser-assisted in situ keratomileusis (LASIK) [6,7]. According to International Dry Eye Workshop (DEWS) II held in 2017, DES was redefined as “Dry eye is a multifactorial disease of the ocular surface characterized by a loss of homeostasis of the tear film, and accompanied by ocular symptoms, in which tear film instability and hyperosmolarity, ocular surface inflammation and damage, and neurosensory abnormalities play etiological roles” [8]. The classification of dry eyes can be categorized into two major subtypes: aqueous deficient dry eye (ADDE), a condition where the lacrimal tear secretion is reduced and hyper evaporative dry eye (EDE), wherein the evaporation of the tear film is uncontrollable with regular lacrimal tear secretion [9,10]. Both these conditions could result in tear hyperosmolarity, which is an important intermediary in causing DES related inflammation. Hyperosmolarity initiates a series of inflammatory events in the epithelial cells which leads to the overexpression of proinflammatory cytokines such as interleukin (IL)-1β, IL-8, IL-6, matrix metalloproteinases (MMPs) and tumor necrosis factor α (TNF-α) on the ocular surface [11,12,13,14]. In addition, inflammation and dryness other symptoms of DES include pain, discomfort, visual hinderance, redness, burning sensation and sensitivity towards light [5].

The first line therapy towards the immediate relief from the clinical symptoms of DES is the use artificial tears (AT) [15]. These are the preferred first course of action to ease the symptoms of DES due to their easy accessibility, cost-effectiveness, low side effect and noninvasive nature. AT provide temporary aid by increasing the moisture content and lubricating the ocular surface, in turn reducing the friction between the eye surface and eyelids. However, artificial tears lack in providing substantial long-term relief from DES related inflammation, which is an important underlying issue that needs to be addressed [16,17]. In several cases, anti-inflammatory drugs have been used to treat the underlying DES related inflammation. Anti-inflammatory agents such as topical corticosteroids alleviates the symptoms and inflammation in DES patients, but its use leads to long-term side effects resulting in cataracts and glaucoma [18]. Very few drugs such as lifitegrast and cyclosporine A have been used to reduce the inflammation associated with DES, however their easy accessibility is still an issue in a few countries [1,19]. Therefore, there is a growing need for an alternative agent for the treatment of DES in terms of both inflammation and other symptoms simultaneously.

Due to the safety concerns pertaining to the use of non-steroidal anti-inflammatory drugs (NSAIDs), there is a growing interest to adopt naturally occurring plant-based products. *Bletilla striata* polysaccharide is a plant-based water-soluble polysaccharide isolated from a terrestrial orchid *Bletilla striata* found in the east Asian countries [20]. Polysaccharides have attained limelight due to their various biologic properties, nontoxicity and biodegradable nature [21]. BSP is known to possess anti-inflammatory, antioxidant and antiviral properties [22,23]. Previous study showed that polysaccharides from *Bletilla striata* can successfully alleviate ROS generation and proinflammatory cytokines activation induced by Angiotensin II in human mesangial cells (HMCs), exhibiting antioxidant and anti-inflammatory properties of BSP [24]. Thus, BSP may play a role as an anti-inflammatory and antioxidant agent for DES treatment. It also exhibits excellent moisturizing and lubricating effect due to its mucoadhesive nature [25]. Therefore, we suspect that BSP-based artificial tears have better therapeutic effect towards DES than AT alone due to the mucoadhesive nature, anti-inflammatory and antioxidant properties of BSP, which would increase the retention time of the eye drop on the ocular surface and simultaneously reduce proinflammatory cytokines and ROS generation on the ocular surface.

The aim of this study was to develop a BPS contained artificial tear with anti-inflammatory property for DES treatment. The feasibility and efficacy of BSP-based artificial tear for DES treatment was examined in vitro by coculturing with inflamed HCECs and by the benzalkonium chloride (BAC)-induced rabbit DES model to evaluate its therapeutic effect in vivo.

## 2. Materials and Methods

### 2.1. Materials

*Bletilla Striata* was purchased from Sheng Chang Company (Taoyuan, Taiwan). Dichlorofluorescin diacetate (DCFDA) kit was obtained from Abcam Company (Eugene, OR, USA). keratinocyte-serum free medium (KSFM), bovine pituitary extract (BPE), insulin, trypsin-ethylenediaminetetraacetic acid (EDTA), penicillin/streptomycin and phosphate-buffered saline were obtained from Gibco BRL (Gaithersburg, MD, USA). Epidermal growth factor (EGF) was purchased from Pepro Tech, Inc. (Rocky hill, NJ, USA). Fibronectin, collagen and albumin (FNC) Coating Mix was purchased from Athena Environmental Sciences, Inc. (Baltimore, MD, USA). live and dead kit, Super Script Ⅲ First-Stand Synthesis System for reverse transcription polymerase chain reaction (RT-PCR), high-capacity cDNA reverse transcription kits, TaqMan Real-Time PCR Master Mixes, Probe, and Trizol were purchased from Thermo Fisher Scientific (Bartlesville, OK, USA). lipopolysaccharide (LPS) was purchased from Sigma-Aldrich (L2880, Rehovot, Israel), Zoletil 50% and 2% Rompun solution were obtained from Virbac Animal Health (Vauvert, Nice, France) and Bayer Korea, Ltd. (Ansan-city, Kyonggi-do, Korea), respectively. Schirmer strips (Tear Touch) were obtained from Madhu Instruments (New Delhi, India). Topical anesthesia solution (0.5% Alcaine1) was obtained from Alcon-Couvreur N.V. (Puurs, Belgium). Fluorescein (FL) paper strips were obtained from HAAG-STREITAG (Köniz/Bern, Switzerland). All the other chemicals were purchased from Sigma-Aldrich.

### 2.2. Extraction of Bletilla Striata Polysaccharide

BSP was extracted by the methods as previously described [26]. Briefly, dry *Bletilla striata* was homogenized and dispersed in 80 °C double distilled water for 4 h, further filtered to remove impurities. The crude extract was precipitated with 3× 95% (*v/v*) ethanol overnight. The resultant precipitate was collected by centrifugation and resolved in distilled water. Deproteinization was carried out by adding 1/3 vol. of chloroform/n-butanol (4:1 *v/v*) and stirred overnight. The above procedure was repeated twice. The final aqueous phase was dialyzed at a molecular weight cutoff of 3000–5000 Da membrane (Orange Scientific, Belgium) and freeze dried to obtain BSP. The structural property analysis of the polysaccharide was performed using Fourier-transform infrared (FTIR) spectrometer (Jasco FT/IR-4200, Tokyo, Japan), ^13^C and ^1^H NMR spectra measurements and TGA-thermogravimetric analyzer (Q50, TA instruments, New Castle, DE, USA). The TGA analysis was performed between 25 and 500 °C with a controlled heating rate at 10 °C/min under a nitrogen atmosphere.

### 2.3. Cell viability of Human Corneal Epithelial Cells (HCECs)

Cell viability assay was performed with live and dead kit (Thermo Fisher Scientific, Bartlesville, OK, USA) according to the manufacturer’s protocol. HCECs (1 × 10^5^/well) were seeded in 24-well plates. After culturing overnight, the medium was replaced with media containing different concentration of BSP (0.01–1% *v/v*). After culturing for one day, the medium containing BSP were discarded and Calcein AM and Ethidium homodimer-1 were added and cell viability was observed using confocal microscope (Olympus IX71, Japan).

### 2.4. Antioxidant Effect of BSP

The ROS content in cells before/after treated by BSP was tested by DCFDA Cellular ROS detection assay kit (Abcam Company, Eugene, OR, USA) according to the manufacturer’s protocol. Briefly, HCECs were seeded in 96-well black plates (2.5 × 10^4^/well) and cultured overnight. The following day, cells were incubated with 200 (μM) H_2_O_2_ for 45 min and subsequently replaced with DCFDA reagent and treated for another 45 min. DCFDA reagent was replaced with medium containing different concentrations of BSP (0.01–1% *v/v*) for 1, 2 and 3 hours, respectively. The amount of 2’, 7’- dichlorofluorescein conversion was determined at 485 nm using a microplate reader (Spectramax plus 384 microplate reader, Molecular devices, CA, USA).

### 2.5. Anti-Inflammatory Effect of BSP by Gene Expression of Inflammatory Cytokines in HCECs

HCECs were seeded in 6-well plate (3 × 10^5^/well). After 24 h incubation, medium was replaced with media containing 500-ng/mL lipopolysaccharide (LPS) for 3 h in order to stimulate inflammation in cells. Unstimulated HCECs were used as control. Further, the medium was replaced with fresh medium containing different concentration of BSP (0.01–1% *v/v*). The cells were collected after 2 h and total RNA was extracted according to the manufacturer’s protocol using TRIzol reagent. The first strand complementary DNA (cDNA) was synthesized from the isolated RNA of different groups stored at −80 °C, with a concentration of 2 μg/μL, respectively, using the high-capacity cDNA Reverse Transcription Kit according to the manufacturer’s protocol. Step One Real-Time PCR System (Applied Biosystems, CA, USA) was used to perform real-time PCR using specific primers (IL-6 [Hs00174131m1], IL-8 [Hs 00174103m1], IL-1β [Hs01555413m1], TNF-α [Hs00174128m1] and TaqMan Universal PCR Master Mix (2×) and glyceraldehyde-3-phosphate dehydrogenase [GAPDH; Hs99999905m1]). The relative expression of each target gene was examined by 2^−△△Ct^ method.

### 2.6. Characterization of Artificial Tears with BPS Addition

First, 0.1% BSP was chosen to be incorporated into the basal AT eye drop solution. The composition of 100 mL basal AT solution consisted of 0.45 g NaCl, 0.015 g CaCl_2_, 0.15 g KCl and 0.45 g Na_2_HPO_4_. The AT solution was freshly prepared and filtered using 0.22 μm filter. In order to characterize the physical properties of BSP-based AT, refractive index was measured using a refractometer (ABBE T3, ATAGO CO.,LTD, Tokyo, Japan), pH and osmolality were measured using a pH meter (Jenco IE2-6171, Shanghai, China) and an osmometer (Osmotech, Advanced instruments, Norwood, MA USA), while the viscosity was measured using a viscometer/rheometer (HAAKE RheoStress 1, Thermo Scientific, Waltham, MA, USA).

### 2.7. Ocular Retention Test for BPS Contained AT

In order to examine the retention time of the eye drops on the ocular surface, two mice (C57BL/6 J) were used in the test (two repeated tests with one-day rest interval). Then, 10 μg/mL red fluorescent dye (TAMRA) was added to only AT or AT-containing 0.1% BSP solution to observe and track the distribution of only AT and BSP-based AT on the eye surface. Five microliters each of only AT and BSP-based AT solution containing fluorescent dye was dropped on the respective mice eye, the fluorescent signal and the photographs were recorded using the In vivo imaging system (IVIS-200 imaging chamber, Xenogen, Alameda, CA, USA) at specific time intervals (5, 10, 15, 30, 45 and 60 min).

### 2.8. Animal Model of DES and Treated by BSP Eye Drops

Eight male New Zealand rabbits (weighing 2.5–3.5 kg) were selected with no indication of ocular inflammation or gross abnormalities. All experimental course of action was in accordance with the institutional animal care and use committee (IACUC) of National Taiwan University College of Medicine and College of Public Health (IACUC approval no. 20170491). The animals were placed in a light-controlled room in standard cages at a temperature of 23 ± 2 °C, with relative humidity of 60% ± 10% and a 12 h light–dark cycle. Animals were fed food and water according to their needs.

All the experimental procedures were conducted under the general anesthesia administered intramuscularly with Zoletil 50 and Rompun (1:2, 1 mL/kg). Then, 20 μL 0.1% benzalkonium chloride (BAC) was instilled three times per day onto the ocular surface of both the eyes of 6 rabbits for four consecutive weeks. The animals were then randomly divided into three groups, with each group containing 4 eyes/2 rabbits and treated with different eye drops: (a) only AT and (b) BSP + AT group containing 0.1% (*v/v*) BSP in AT solution and (c) 0.1% (*v/v*) BAC eye drops used as negative control. The fourth group consist of 2 more rabbits (4 eyes) without BAC inducement which were treated as positive controls. These eye drops were instilled onto the ocular surfaces of rabbits for three weeks, thrice a day at regular intervals. After monitoring for three weeks, the appearance of the ocular surface of rabbits was observed via bright field, slit-lamp and fluorescein staining. To further confirm the treatment effect, Schirmer’s test and cornea thickness tests were also performed. Thereafter, the rabbits were euthanized, and corneas were excised for histological examination. All the above tests are described below.

#### 2.8.1. Measurement of Tear Production

Schirmer strips were used to measure the aqueous tear secretion [27]. Briefly, anesthesia was administered to the rabbits to keep them motionless. The procedure was conducted in a standard environment by the same person at defined time points. Post topical administration of 0.5% Alcaine^®^, Schirmer strips were placed by pulling the lower eyelid on the palpebral conjunctival vesica, which is located near the intersection of the middle and outer third of the lower eyelid. The wetted length of the strip was measured (in millimeters) after 5 min. The average length of the wetted study was calculated by testing each eye twice at an interval of 30 min.

#### 2.8.2. Central Corneal Thickness Measurement (CCT)

Using an ultrasonic pachymeter (iPac® pachymeter, Reichert Technologies, Depew, NY, USA) with a hand-held solid probe, cornea thickness was measured. The tip of the probe was held perpendicular to the center of the cornea. Three readings were recorded for each eye and the average was calculated.

#### 2.8.3. Appearance of the Ocular Surface

The ocular surface of the rabbits of each group were observed via slit-lamp microscope after fluorescein staining. For fluorescein staining, 1% fluorescein sodium (2 μL) was dropped onto the ocular surfaces of the rabbits and observed with a hand-held slit-lamp (SL-17, Kowa Company, Ltd., Torrance, CA, USA).

#### 2.8.4. Histological Examination of the Cornea

After euthanizing the rabbits, corneas were fixed for 24 h in 3.7% formaldehyde solution. The fixed corneas were then embedded in paraffin and sectioned. The sections were then stained with hematoxylin and eosin (H&E) and observed under a microscope.

## 3. Results

### 3.1. Characterization of BSP

**Figure 1:**
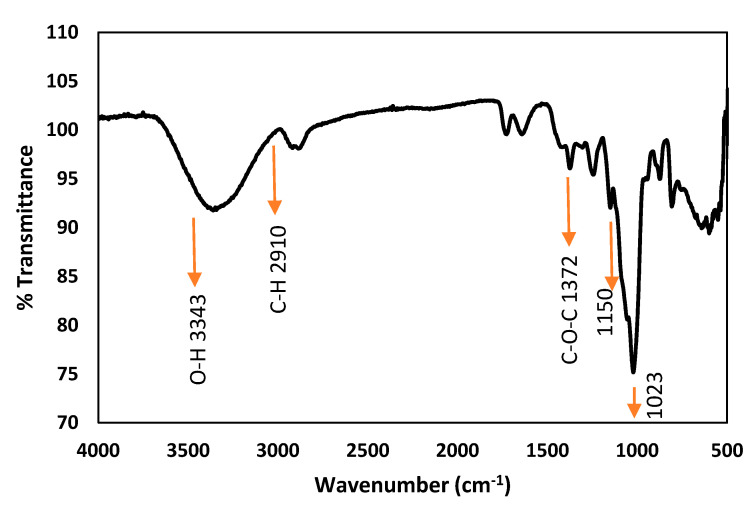
FTIR characterizations of *Bletilla striata* polysaccharide (BSP).

After the extraction from the herbal orchid, the identification results of BPS by FITR, TGA, and NMR is provided here. For FTIR spectra, the characteristic absorption at 874 cm^−1^ corresponds to the β-glucosyl residues. The absorption peak at 809 cm^−1^ suggested that BSP contains mannose while the peaks at 1023 and 1150 cm^−1^ revealed the extant of pyranoses (Figure 1). The present FTIR results are fully in consistence with previous reports [20,26].

**Figure 2:**
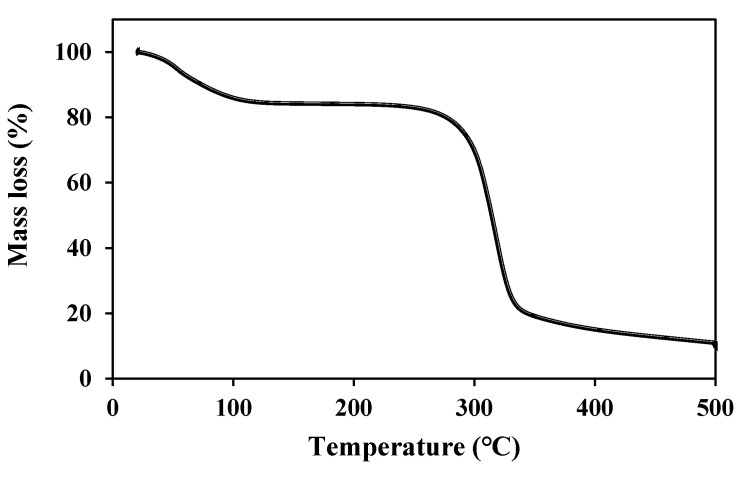
Thermal characterization of BSP (TGA).

For thermal decomposition of BSP, TGA curve was obtained. There are two significant weight losses (Figure 2). The first mass loss is at 100 °C corresponding to the loss of adsorbed water of the sample while the second mass loss is at the onset of 250 °C corresponding to the unique characteristic feature of BSP. These results are also in consistent with the previous reports [28].

**Figure 3:**
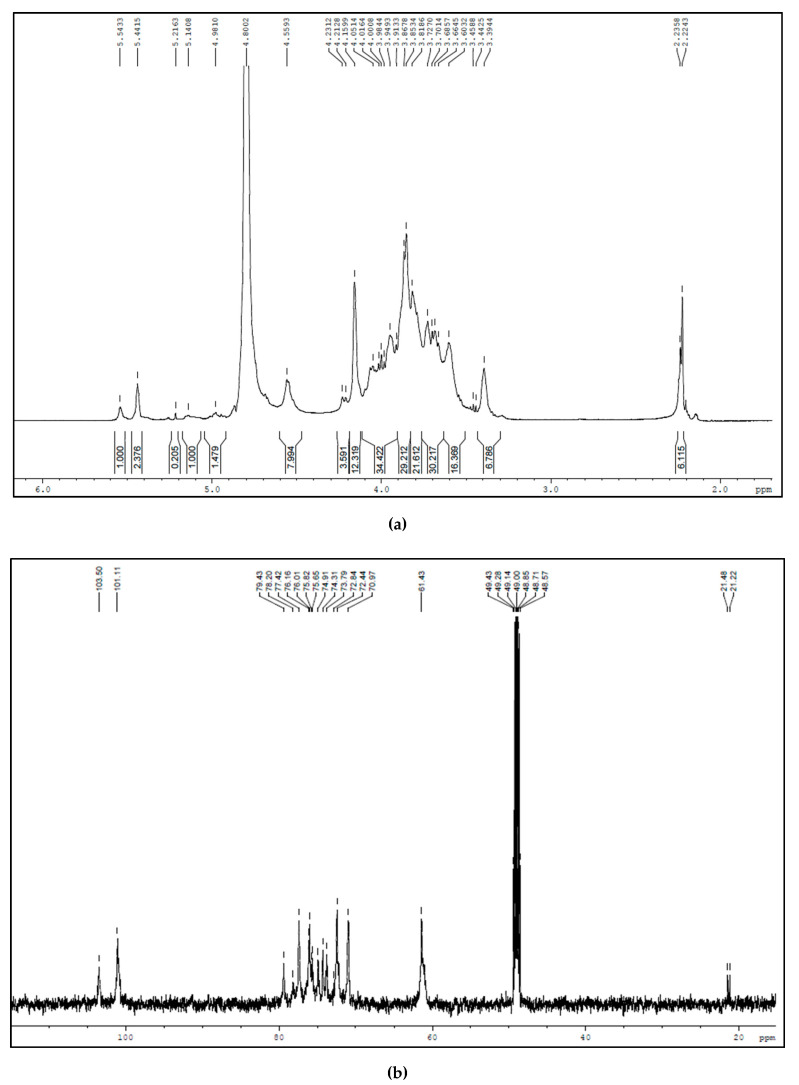
(**a**) ^1^H NMR spectrum of BSP; (**b**) ^13^C NMR spectrum of BSP.

The structure of BSP was analyzed by ^1^H and ^13^C NMR spectroscopy. The ^1^H NMR spectra showed two main peaks at δ 4.80 and δ 4.6 ppm (Figure 3a). The chemical shift of carbohydrate indicates the signals for anomeric protons (R). The uncharacteristic peaks found between 3–4 ppm indicates the non-anomeric ring protons. For better elucidation, ^13^C NMR was performed and the spectra showed two main peaks at δ103.50 and δ100.08 ppm (Figure 3b), revealing the existence of β-glucopyranose and α-mannopyranose repeating units in BSP (R). The chemical shifts found in ^13^C NMR further confirmed the anomeric carbon. The ^1^H NMR and ^13^C NMR chemical shifts were similar to that of an ion exchange chromatography purified BSP [29].

### 3.2. Cell Viability of HCEC

**Figure 4:**
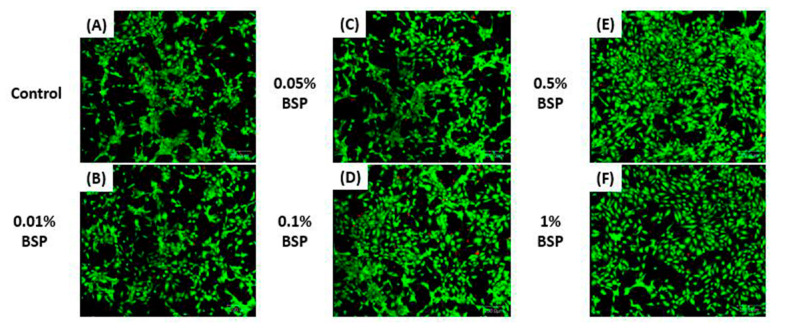
Cell viability of human corneal epithelium cells (HCECs) assessed by live and dead staining (**A**) Control group, HCEC treated with (**B**) 0.01% BSP; (**C**) 0.05% BSP; (**D**) 0.1% BSP; (**E**) 0.5% BSP; (**F**) 1% BSP; scale bar: 200 µm.

The effect of different concentrations of BSP (0.01–1%) on HCECs was demonstrated by live and dead staining (Figure 4). The result shows that HCECs has good cell viability with all the given concentrations of BSP proving the biocompatibility of BPS.

### 3.3. Antioxidant Effect of BSP by DCFDA Cellular ROS Detection Assay

**Figure 5:**
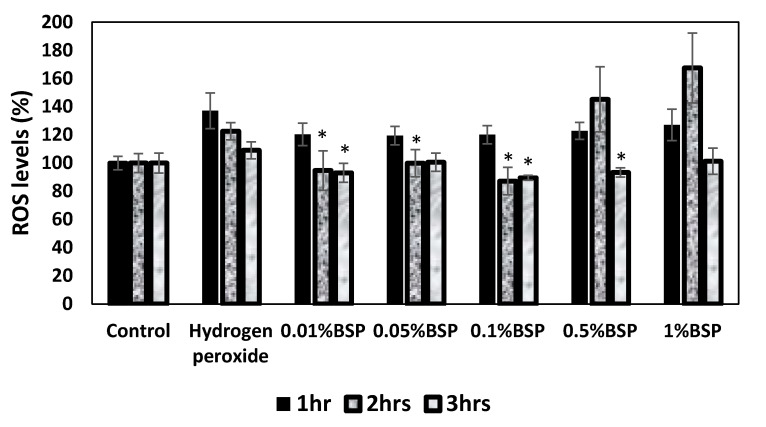
DCFDA cellular R reactive oxygen species (ROS) OS detection assay (n = 6), * *p* < 0.05 compared to hydrogen peroxide group by one-way ANOVA.

The DCFDA experiment was used to confirm the antioxidant effect of BSP by quantifying the ROS level in HCECs (Figure 5). It was demonstrated that the lower concentrations of BSP (0.01%, 0.05% and 0.1%) can reduce the ROS levels more effectively than the higher concentrations (0.5% and 1%). Therefore, BSP-based AT would be able to reduce the oxidative stress by scavenging ROS on the ocular surface of DES model.

### 3.4. Anti-Inflammatory Effect of BSP by Gene Expression of Inflammatory Cytokines in HCECs

**Figure 6:**
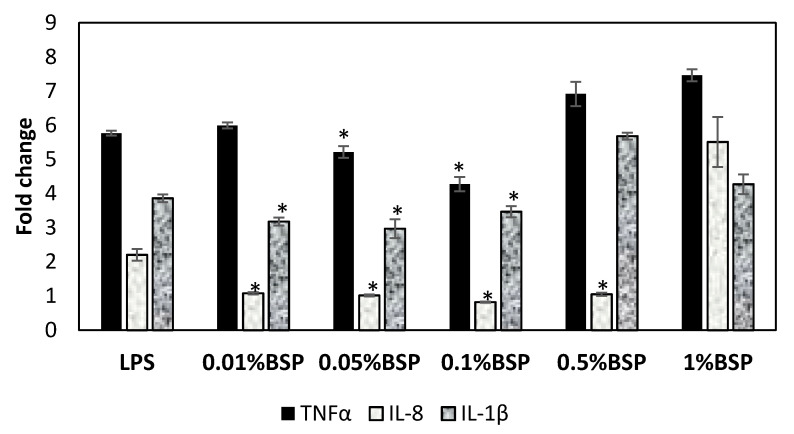
Expressions of inflammatory cytokine (TNF)α, IL8 and IL1β in lipopolysaccharide (LPS)-induced HCEC following different concentrations of BSP treatment. Cells not treated with LPS were used as controls. (n = 3), * *p* < 0.05 compared to LPS group by one-way ANOVA.

Anti-inflammatory effect of BSP was examined by measuring the levels of the proinflammatory cytokines TNFα, IL8 and IL1β in inflammation stimulated HCECs incubated with LPS and BSP with concentrations 0.01%, 0.05%, 0.1%, 0.5% and 1%. In Figure 6, the expressions of TNFα, IL8 and IL1β were upregulated post LPS inducement in HCECs, which was expected, as LPS treated cells replicates the inflammatory condition associated with DES. However, expressions of TNFα, IL8 and IL1β significantly decreased in cells treated with 0.05% and 0.1% than the cells treated with LPS (* *p* < 0.05). Further, IL8 and IL1β expressions were downregulated in cells treated with 0.01% BSP than in cells treated with LPS (* *p* < 0.05). The overall result shows that the lower concentrations of BSP (0.01%, 0.05% and 0.1%) provide better anti-inflammatory effect by significantly relieving the inflammation in HCECs. Therefore, in view of the above results, 0.1% BSP was chosen to be an optimal concentration for further experiments in the animal model.

### 3.5. Physical Properties of Artificial Tears

Table 1 shows the summary of the physical properties between human tears [30,31,32] and BSP artificial tears. The viscosity (4.6–4.7 mPa s) and osmolality (266 ± 0.82 mOsm/kg) of BSP-based AT is within the range of normal human tears, while the refractive index of AT+BSP is 1.3345 ± 0.0015, which is very close to that of real human tears. However, the pH value of artificial tears (7.68 ± 0.01) is slightly out of range when compared to the real human tears, but it is still acceptable.

### 3.6. Effect of BSP to Enhance the Retention of AT on the Ocular Surface

**Figure 7:**
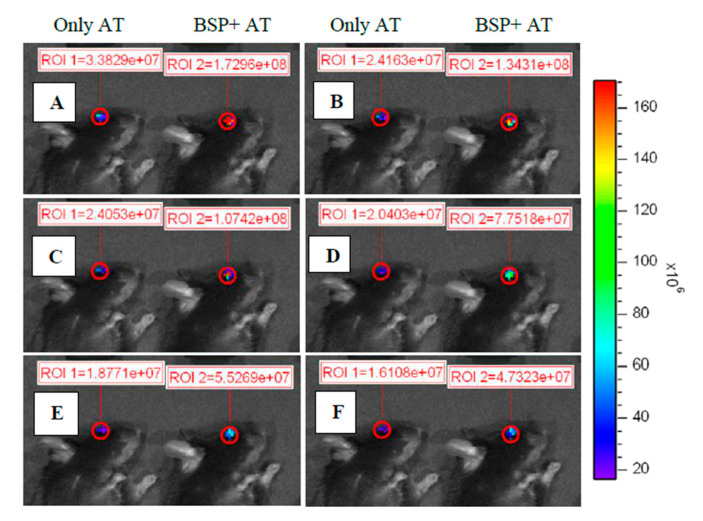
Color photographs showing the ocular retention of the artificial tears mixed with dye (TAMRA) on the ocular surface of mice treated with only artificial tears (AT) and BSP + AT after (**A**) 5-min; (**B**) 10-min; (**C**) 15-min; (**D**) 30-min; (**E**) 45-min and (**F**) 60-min exposure to the artificial tears.

A florescent dye (TAMRA) was mixed with AT-containing BSP (BSP+AT) and only AT in order to detect the florescent signal via IVIS to observe its distribution in the mice’s eyes. Following 5-min, 10-min, 15-min, 30-min, 45-min and 60-min exposure to the respective eye drops in Figure 7, mice treated with BSP + AT exhibited higher fluorescent intensity than mice treated with only AT. This result indicated that BSP can indeed increase the retention time of the artificial tears and impart lasting lubrication onto the ocular surface, due to the mucoadhesive nature of BSP.

### 3.7. Appearance of the Ocular Surface

**Figure 8:**
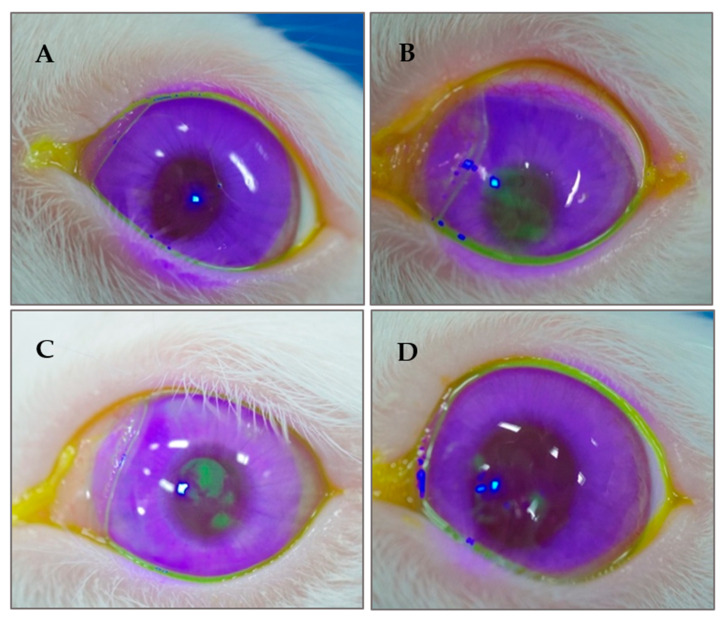
Slit-lamp images of the ocular surface of rabbits after fluorescence staining. (**A**) Control eye; (**B**) eye treated with 0.1% BAC (negative control; dry eye syndrome (DES) eye); (**C**) eye treated with only AT and (**D**) eye treated with AT-containing 0.1% BSP.

Figure 8 shows the appearance of the ocular surface under a slit-lamp microscope, examined three weeks post the treatment with different eye drops. The ocular surface of the positive control group did not exhibit any fluorescent staining when observed under a slit-lamp microscope. In contrast, BAC eye drops treated group (negative control) exhibited many fluorescent residues on the corneas of the rabbits, indicating inflammation induced by BAC onto the ocular surface. Few staining residues were observed in the corneas treated with only AT eye drops. However, no staining residues were observed in BSP + AT group.

### 3.8. Changes in Tear Production

**Figure 9:**
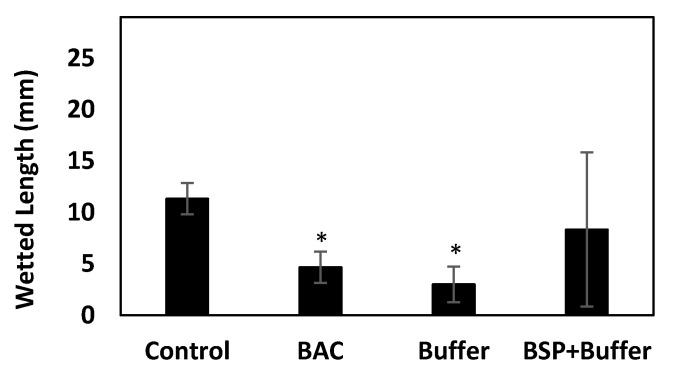
Tear secretion acquired by Schirmer’s test (n = 3), * *p* < 0.05 compared to control group by one-way ANOVA.

Tear production is an important indicator for DES evaluation. DES-induced rabbits were examined three weeks after the treatment with different eye drops (Figure 9). Compared to the average wetted length of the positive control group (no DES) (11.33 ± 1.53 mm), wetted average length of BAC group (replicating DES) was 4.7 ± 1.53 mm, indicating decreased tear secretion in the BAC group. The tear production in only AT group had no significant difference (* *p* < 0.05) compared to the BAC group. However, the average wetted length of BSP + AT group was recorded to be 8.3 ± 7.50 mm, indicating increased tear production in BSP treated group than the BAC group.

### 3.9. Changes in Cornea Thickness

**Figure 10:**
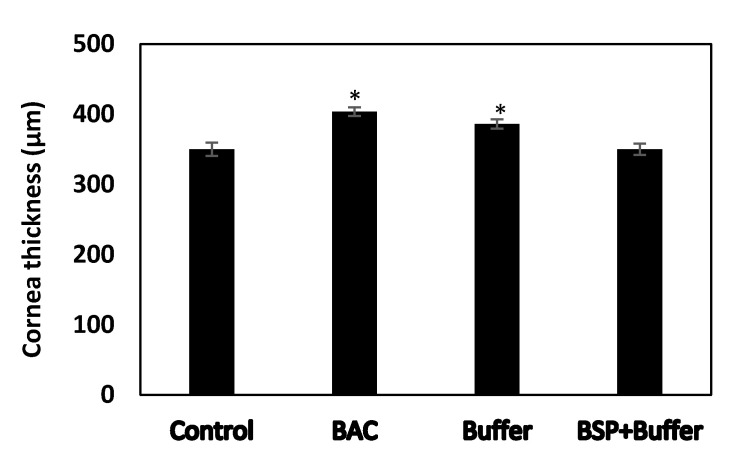
Variation of cornea thickness in rabbits (n = 8), * *p* < 0.05 compared to control group by one-way ANOVA.

Cornea thickness was measured post three weeks after the treatment with different eye drops. Compared to the positive control group showing average cornea thickness of 349.75 ± 9.49 μm, the average cornea thickness of BAC group increased and was found to be 403.75 ± 6.18 μm, indicating inflammation and swelling of the tissue. The average cornea thickness of only AT group did not show any significant difference compared to the BAC group. However, the average cornea thickness of BSP + AT group was recorded to be 350 ± 8.02 μm, which was similar to the normal cornea thickness (Figure 10).

### 3.10. Histological Examination of the Cornea

**Figure 11:**
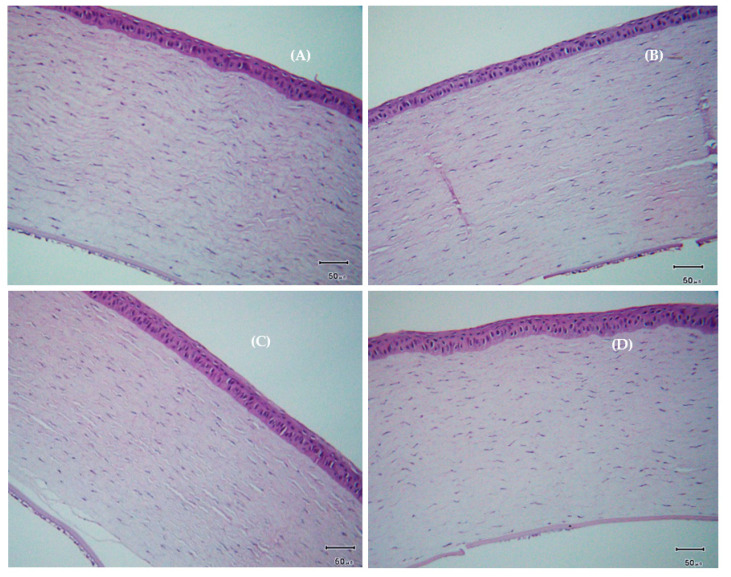
Histological section of cornea with H&E staining acquired from (**A**) control; (**B**) benzalkonium chloride (BAC); (**C**) only AT and (**D**) BSP + AT treated groups, scale bar: 50 µm.

Examination under a light microscope showed multilayered (three to five layers) epithelial cells with an average thickness of 37.85 ± 3.19 µm and a dense stromal layer containing collagen fibrils in the corneas of the control group (Figure 11A). Defects were seen in rabbits of BAC group (mimicking DES) as it had thinner corneal epithelium consisting of one or two layers with an average thickness of 24.28 ± 2.99 µm (Figure 11B). The only AT group (Figure 11C) and BSP + AT group (Figure 11D) had no significant difference between the corneal epithelium layer, however the stromal layer in BSP + AT treated group was dense than the AT group alone. The tear volume was increased in group treated with BSP + AT than the only AT group indicating therapeutic effects of BSP-based artificial tears on dry eye model.

## 4. Discussion

Artificial tears have gained importance because of their ability to provide immediate ocular comfort in DES. However, most of the available artificial tears fail to address the underlying issue of DES related inflammation. This study confirmed the mucoadhesive and anti-inflammatory effect of BSP-based artificial tears towards effective treatment of DES in rabbit model.

We found that BSP showed a good biocompatibility and was non-toxic to HCECs (Figure 4). This finding was in accordance with the previous study by Wu *et al*., who showed that BSP had a positive effect on the proliferation of HCECs and demonstrated no cytotoxicity. Dry eye related inflammation is mediated by pro-inflammatory cytokines and inflammatory markers. The most widely studied pro-inflammatory cytokine accompanying dry eye is Interleukin (IL)-1 alongside IL-6, IL-8, IL-1β and TNFα which is also known to play a significant role in DES-related inflammation [33]. These cytokines and inflammatory markers are secreted by lacrimal gland and start the inflammatory reactions causing damage to the epithelial cells on the ocular surface along with the reduction of mucin producing goblet cells causing further damage to the surface [12]. In view of the existing literature, BSP is able to control the levels of pro inflammatory factors in wound healing, thereby suppressing inflammation at the site [34]. Similar results were observed in inflamed HCECs treated with different concentrations of BSP. Lower concentrations of BSP (0.05% *v/v* and 0.1% *v/v*) were able to downregulate the expressions of TNFα, IL8 and IL1β in HCECs (Figure 6), confirming its anti-inflammatory property, which would be beneficial in suppressing DES related inflammation.

Human eye tends to be exposed to oxidative stress due to its high metabolic activity and elevated oxygen tension [35]. Several antioxidants such as uric acid, ascorbic acid, cysteine, lactoferrin present in the tear fluid help protect the ocular surface against certain radicals such as hydroxy and peroxyl radicals [36]. Instability in the tear film due to DES can cause over production of ROS on the ocular surface [37]. Therefore, scavenging ROS is necessary to protect the ocular surface from injury related to oxidization. According to the previous reports, BSP exhibit antioxidant properties and has an ability to scavenge the free radicals, [38]. In this study, DCFDA assay showed that 0.01%, 0.05% and 0.1% BSP can effectively reduce the ROS levels (Figure 5), confirming its antioxidant property, which would help reduce the oxidative stress in DES models.

Mucoadhesion plays a key role in formulating a successful candidate for ocular delivery [39]. BSP is proven to be a mucosa protective agent [25]. The mucoadhesive property of BSP helps to form a protective film on the ocular surface thereby, promoting lubrication and moisturization of the ocular surface, eventually reducing the friction while blinking. In this study, mice were used to demonstrate the retention time and distribution of BSP eye drops on the ocular surface. Mice were used instead of rabbits due to their smaller size which can easily fit in the restricted space of the imaging chamber. Higher fluorescent intensity was observed on the ocular surface of the mice treated with BSP + AT eye drops than ocular surface treated with AT alone (Figure 7) at specific exposure points, indicating BSP’s longer retention time on the ocular surface, thereby, providing lasting lubrication and enabling symptomatic relief.

Rabbit animal model is preferred for conducting ophthalmic experiments due to the larger surface area of their exposed ocular surface, making it easily accessible for performing clinical tests like Schirmer’s test and fluorescence staining. According to the previous studies, BAC is used to induce DES in animal model [27,40]. However, BAC is also used as a preservative in many ophthalmic eye drops and is shown to aggravate the pre-existing dry eye by causing inflammation and damage to the ocular surface and disturbing the tear film stability [41]. However, inflammation and tear film instability are also a common characteristic of DES, therefore in this study, BAC is used to induce DES in rabbit animal model.

Improvements were observed in recommencing tear volume and cornea thickness in the DES rabbits treated with BSP-based AT. Tear production increased 1.75-fold in rabbits treated with BSP + AT (Figure 9) due to the mucoadhesive nature of BSP that promotes water retention capacity onto the ocular surface. The cornea thickness reduced in the rabbit eyes treated with BSP + AT than the DES-induced eyes (Figure 10) due to the enhanced therapeutic effect of BSP eye drops in reducing swelling and inflammation. Additionally, our study provides the values of refractive index (1.3345 ± 0.0015), pH value (7.68 ± 0.01), viscosity (4.6–4.7 mPa s) and osmolality (266 ± 0.82 mOsm/kg) of BSP-based artificial tears (Table 1), which are all very close to the composition of natural human tears, validating its suitability as a substitute to treat DES. In the present study, H&E examination showed damage to the corneal epithelium (2–3 layers) post BAC inducement (Figure 11B). BAC being a small molecule penetrates through the epithelial layers deep into the stromal layer causing keratocyte damage. The epithelial layers were regained, and stroma was realigned post treatment with AT-containing BSP (Figure 11D).

This study reveals the advantages of using BSP to reduce inflammatory genes expression in inflamed HECEs in vitro, and also to relieve other DES symptoms by recommencing the tear volume, increasing the water retention capacity and promoting corneal recovery in DES model in vivo.

## 5. Conclusions

The BSP was successfully extracted and formulated into an artificial tear solution for DES treatment. Different concentrations of BSP (0.01–0.1%) cocultured with HCECs showed no toxicity in HCECs. The 0.01%, 0.05% and 0.1% BSP exhibited good antioxidant capacity by lowering the ROS level effectively in stimulated HECEs. While 0.05% and 0.1% BSP demonstrated a good anti-inflammatory effect by significantly downregulating the expression of TNFα, IL8 and IL1β in inflamed HCECs. Therefore, according to the above results, 0.1% BSP was chosen to be the optimal concentration to be mixed with the artificial tear solution for further tests in DES-induced rabbit’s model. The BSP artificial tears mimics the natural human tears in terms of similar osmolality, pH, refractive index and viscosity. BSP in AT increased the retention on the ocular surface. The administration of AT-containing BSP effectively promoted corneal recovery and improved tear secretion in DES-induced rabbits to some extent. H&E result shows the recovery of multilayered epithelial cells and stromal layer in corneas of DES rabbits treated with BSP artificial tears. The overall findings of this study suggest that the BSP-based artificial tears not only impart symptomatic relief by providing a good moisturizing and lubricating effect but is also beneficial in providing long term relief from DES-related inflammation which is an underlying issue in the treatment of DES. The present study shows the potential effect of BSP as a promising therapeutic candidate in the treatment of DES. Further clinical examinations are required to evaluate the safety and efficacy of the formulated BSP eye drops for the use in humans to treat DES.

## Figures and Tables

**Table 1:** Characteristics of human tears [30,31,32] and artificial tears.

Refractive Index		pH Value	Viscosity (mPa s)	Osmolality (mOsm/kg)
Human tears	1.337 (Approx.)	6.5–7.6	1–10	260–340
0.1% BSP+AT	1.3345 ± 0.0015	7.68 ± 0.01	4.6–4.7	266 ± 0.82
